# Shear wave velocity prediction using Long Short-Term Memory Network with generative adversarial mechanism

**DOI:** 10.1371/journal.pone.0325271

**Published:** 2025-06-24

**Authors:** Xingan Fu, Youhua Wei, Yun Su, Haixia Hu, Ji Zhang, Quan Wang

**Affiliations:** 1 College of Mathematics and Science, Chengdu University of Technology, Chengdu, China; 2 Geomathematics Key Laboratory of Sichuan Province, Chengdu University of Technology, Chengdu, China; 3 Chengdu North Petroleum Exploration and Development Technology Company Limited, Chengdu, China; The University of Texas, MD Anderson Cancer Center, UNITED STATES OF AMERICA

## Abstract

Shear wave velocity (Vs) serves as a crucial petrophysical parameter for subsurface characterization, yet its acquisition remains challenging. While long short-term memory (LSTM) networks have emerged as the predominant solution for Vs prediction by synthesizing contextual relationships among conventional logging curves, existing implementations often overlook characteristic discrepancies between training and prediction datasets, leading to suboptimal performance. This study proposes an enhanced LSTM architecture integrated with a generative adversarial mechanism (LSTM-GAM) to address this limitation. The framework employs a dual-component structure: 1) A primary LSTM backbone that captures contextual dependencies across multi-logging sequences, and 2) An adversarial module where the generator minimizes reconstruction errors while the discriminator identifies essential feature representations common to both training and predictive data. This synergistic architecture not only preserves sequential correlations but also enhances cross-domain adaptability through adversarial feature alignment. We validate the model’s efficacy using logging data from two vertical wells in the South China Sea. Comparative experiments demonstrate the proposed LSTM-GAM achieves superior prediction accuracy with a mean absolute error (MAE) of 59.4 m/s and determination coefficient (R²) of 0.9064, outperforming conventional LSTM network. Further ablation studies reveal consistent performance improvements across varied input configurations, confirming the method’s enhanced generalization capability for Vs prediction tasks. The technical advancement provides an effective data-driven solution for shear wave velocity estimation in complex geological environments.

## 1. Introduction and literature review

### 1.1 Introduction

**Fig 1:**
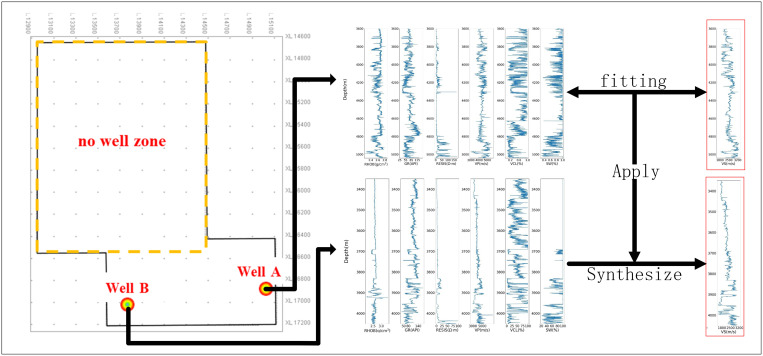
General diagram of shear wave predicting.

Shear wave velocity is a crucial geophysical parameter for studying reservoir AVO (Amplitude Versus Offset) characteristics, playing a significant role in the evaluation of underground reservoirs [1,2]. When the velocity can be accurately determined within a certain depth range in certain wells, it greatly aids in the detection of oil deposits and enhances the oil drilling with lower risk and optimal strategy [3]. However, there is a severe conflict between the availability of shear wave velocity because of various reasons (expensive core sampling testing and unavailable dipole acoustic logging tools et al) [4,5]. Moreover, the petrophysical approach, which involves making an equivalent medium replacement based on the physical properties of underground rocks and calculating the corresponding theoretical P- and S- wave velocities [6], faces limitations. The accuracy of this method heavily relies on the availability of precise petrophysical parameters, including porosity, pore shape, and mineral composition [7]. Unfortunately, these parameters are often difficult to obtain or are subjectively set, which compromises the accuracy of shear wave velocity predictions using petrophysical methods. Consequently, petrophysical analysis tends to depend excessively on specific geological conditions and the prior expertise of professionals, limiting its broader applicability. Given these challenges, there is a pressing need to develop high-precision methods for predicting shear wave velocity to enhance the accuracy of underground reservoir exploration. A promising approach involves synthesizing shear wave velocity data from remaining conventional logging curves (Fig 1), which could provide a more reliable and accessible means of obtaining this vital parameter.

### 1.2 Literature review

Shear wave velocity is intrinsically related to the matrix mineral type, rock porosity, fluid inclusions, and environment of underground rock formations [8–10]. Since both P-wave velocity and S-wave velocity are influenced by these factors and exhibit a strong correlation, many researchers use P-wave velocity to regress the empirical formula of S-wave velocity based on data statistics [6,9,10–13]. For instantly, Pickett and Castagna established empirical relations for estimating shear wave velocities in limestone and clastic rocks, respectively [9,14]. While the empirical formula is a fast and convenient VS estimation method, the generalization and accuracy of this method are limited due to varations in rock types and geographical locations [15]. Furthermore, to improve prediction accuracy, it is essential to incorporate additional logging curves related to shear wave velocity, which necessitates innovation in modeling approaches. For example, Akhundi achieved a lower prediction error by including porosity and density logging, and many researchers have attempted to integrate more logging parameters to enhance the accuracy of S-wave velocity predictions (Anemangely, 2017; Dvorkin, 2008) [16,17]. The essence of the problem lies in identifying the influencing factors of S-wave velocity and accurately inferring its true value [3]. With the development of technology, machine learning has emerged as a powerful tool for geological parameter estimation [18–21]. For instance, Bagheripour et al. (2015) and Anemangely et al. (2019) utilized support vector regression models (SVR) to predict Vs and achive a higher accuracy compared to traditional empirical formulas [22,1]. The SVR algorithm works by mapping features into high-dimensional space and establishing hyperplanes, enabling the separation and recombination of primary features for predicting [23]. To simplify implementation and get high accuracy, Nourafkan and Kadkhodaie-Ilkhchi (2015) employed a fuzzy inference system to predict the velocity, resulting a strong robustness model [24]. Similarly, Wang and Peng (2019) applied the Extreme Learning Machine (ELM) to predict Vs, achiving a model with high computational efficiency [25]. Although traditional machine learning can achieve good numerical fitting and representational accuracy, it may struggle to capture complex or implicit relationships within the data, limiting its sustainability in more intricate scenarios.

Moreover, compared to the low-level and figurative features learned by traditional machine learning methods, deep neural networks are more capable of establishing profound mappings between inputs and outputs. Deep learning excels at extracting key information from the deep level of variables relations and has made great achievements in speech recognition, natural language processing, and reinforcement learning [26]. Kheirollahi et al. (2023) used standard logging curves as inputs of ANN to predict the shear wave velocity in carbonate formations, achieving a lower RMSE error compared to linear regression and ensemble learning [27]. Additionally, Zhang et al. (2018) employed LSTM to approximate missing logging data, resulting in more realistic and usable data [28]. Similarly, Jun Wang et al. (2020) and Jiachun You et al. (2021) incorporated the background information of logging data with LSTM network, ultimately achieving superior results of VS prediction compared to ANN [7]. The LSTM-based models exhibit strong learning capabilities for time-series data, effectively mitigating the risk of overfitting. Moreover, Chen et al. (2022) integrated the feature extraction of CNN into LSTM network, achieving a higher accuracy than the original model [29]. The three thresholds of the LSTM or GRU network greatly increase the ability of the network to assimilate contextual information using adjacent multiple samples, thus effectively improving the precision [30]. Notably, LSTM network demonstrates remarkable computational efficiency through parallel cells. Because its cells have long-term memory ability, LSTM network can retain key effective information and deal with nonlinearity caused by time variability [31]. In summary, the LSTM network exhibits excellent performance in sequence processing [32].

A comprehensive review of the relevant literature reveals that the neural network has the highest accuracy among current ways for Vs prediction (Table 1). While various advanced techniques such as board learning systems and integrated LSTM approaches make a lot of contributions to enhancing model performance, they are still not involved in the feature discrepancy between the trained and predicted data. Summarily, two main challenges remain unresolved:(1) Precision challenge: how to design an overall network capable of representing the S-wave velocity mapping model across diverse geological formations; (2) Generalization challenge: how to mitigate feature differences between training and prediction to eanhance the adaptability of models. To address these dual challenges, this paper proposes a novel framework combining long short-term memory networks with a generative adversarial mechanism. The proposed architecture employs LSTM as its core component for contextual modeling and feature extraction. The features extracted by the LSTM network are subsequently processed by a generator to produce enhanced representations for both training and prediction phases. Finally, a discriminator is employed to classify these features’ origins and progressively minimize the distribution gap between training and prediction. Through this innovative approach, the accuracy of shear wave velocity is substantially improved, thereby facilitating more precise oil and gas exploration.

**Table 1:** the main structural differences among different algorithms.

	Feature extraction	Applicable scenario	Usage
ER	Fixed formula (single variable)	The regional geological characteristics are simple, and the changes are not drastic	Formula setting and data fitting
MR	Fixed formula (multiple variables)	Multi-factor control and linear correlation	Formula setting and data fitting
SVM	Hyperplanes which are partitioned by Polynomial or kernel density functions	Non-artificial feature design, which mainly focuses on numerical features, belongs to shallow feature extraction	Design polynomial forms or density functions to train to iteratively fit
ANN	Extracted by features of different layers	Be suitable for the case of possessing complex relationships and relatively independent samples	The layered structure of neurons is designed and decrease the value of the loss function
LSTM	Variable features with considering context from samples of a certain length	Extracting the background features of the current samples, which is suitable for fitting trends	Setting the input sample length, number of neuron layers, and backpropagate until the loss is minimized

The organizational structure of this paper is as follows. First, we present the theoretical methodologies employed in this study, including Artificial Neural Networks (ANN), Long Short-Term Memory (LSTM), and the novel GAN-LSTM framework proposed herein. Subsequently, we detail the logging dataset utilized, outline the workflow of the proposed algorithm, and define the evaluation metrics. Next, we train the GAN-LSTM network using measured log data and compare its predictive accuracy with several methods discussed in the literature review. Finally, we assess the prediction performance of our approach under varying input length parameters. Experimental results demonstrate the proposed model achieves superior outcomes, highlighting its effectiveness in shear wave velocity estimation.

## 2. Method and theory

### 2.1 Artificial Neural Network (ANN)

**Fig 2:**
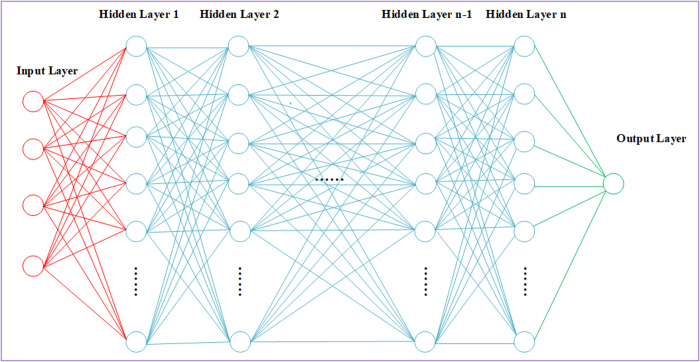
ANN structure diagram.

The artificial neural network (ANN) comprises three essential components: an input layer, hidden layers, and an output layer [33]. In this framework, well log parameters correlated with shear wave velocity are fed into the input layer, followed by the establishment of complex mapping relationships between the log data and shear wave velocity through the hidden layers, ultimately yielding predictions at the output layer (Fig 2). Adjacent hidden layers relate to weights, complemented by activation functions to introduce non-linearity. The final hidden layer extracts characteristic variables through a linear transformation process. To approximate predicted value with actual measurements, a loss function is implemented to quantify the discrepancy between predicted and observed values. Through iterative optimization, the trained model becomes a predictive tool for shear wave velocity estimation.

The formula in two adjacent layers of the network can be expressed as:


$$Y_{j}^{l}=\sigma (\sum (W_{ij}^{l}X_{i}^{l-1})+b_{j}^{l})$$ (1)

Where, $Y_{j}^{l}$ on behalf of the *j* neuron of the *l* layer, $X_{i}^{l-1}$ refer to the *i* neuron of the *l* − 1 layer, $W_{ij}^{l}$ is the weight matrix of the *l* layer, $b_{j}^{l}$ represents the corresponding bias, and the sigmoid activation function is represented as σ.

### 2.2 Long Short-term Memory Network (LSTM)

**Fig 3:**
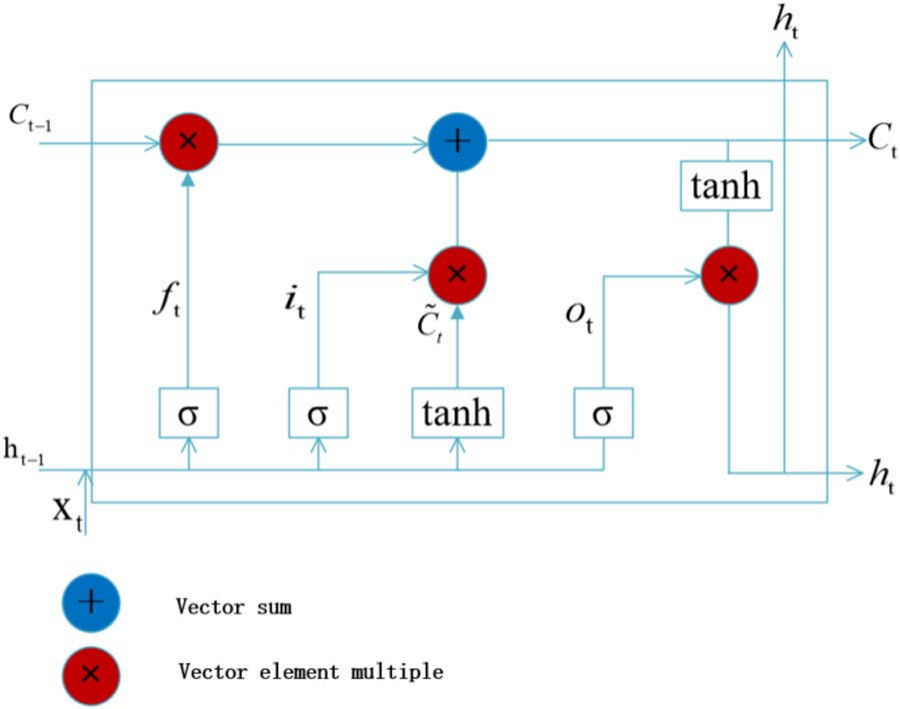
LSTM network cell internal structure.

The LSTM (Long Short-Term Memory) network, introduced by Hochreiter and Schmidhuber [34], enhances the traditional ANN by transforming hidden layer information into a format that can propagate along the sequence direction. The LSTM network consists of multiple LSTM units (Fig 3), which are used to build the mapping relationship of each sequence information. Each unit incorporates three gate structures, which are used to select the hidden layer information of the mapping relationship from the previous sequence giving into the next hidden layer of the following sequence. The input gate identifies the valuable information to participate into the calculation; The forget gate control discards irrelevant information from the previous time step; The output gate determines which hidden layer information should be passed to the subsequent unit. By leveraging these mechanisms, the LSTM network provides a robust theoretical foundation for extracting sequential features from logging curves, making it highly effective for shear wave prediction. Unlike traditional ANN models, which focus solely on current input variables, the LSTM-based prediction model captures contextual information across sequences, enabling a more comprehensive understanding of the data. This capability makes the LSTM network particularly advantageous for extracting sequential patterns, thereby improving the accuracy and reliability of predictions.

The unit of LSTM includes input gate (***i***_***t***_), forgetting gate (***f***_***t***_), status update layer (***c***_***t***_), output gate (***o***_***t***_), hidden layer (***h***_***t−1***_) at *t-*1 time, hidden layer ***h***_***t***_ at *t* time, and input ***x***_***t***_ at *t* time. The calculation of LSTM cell can be expressed as the following formulas:


$$i_{t}=\sigma (W_{i}[x_{t},h_{t-1}]+b_{i})$$ (2)

$$f_{t}=\sigma (W_{f}[x_{t},h_{t-1}]+b_{f})$$ (3)

$$C_{t}=f_{t}*C_{t-1}+i_{t}*\tanh(W_{c}[x_{t},h_{t-1}]+b_{c})$$ (4)

$$o_{t}=\sigma (W_{o}[x_{t},h_{t-1}]+b_{o})$$ (5)

$$h_{t}=o_{t}*\tanh(C_{t})$$ (6)

Where ***W***_***i***_**, *W***_***f***_**, *W***_***c***_**, *W***_***o***_ and *b*_*i*_, *b*_*f*_, *b*_*c*_, *b*_*o*_ are the weight matrix and bias term of the input gate, the forgot gate, the update gate, and the output gate respectively. “σ” and “tanh” are sigmoid and tanh activation functions, respectively; “ * ” expression dot product operation; [**a**, **b**] indicates that the tail of vectors **a** is connected to the head of vectors **b**.

**Fig 4:**
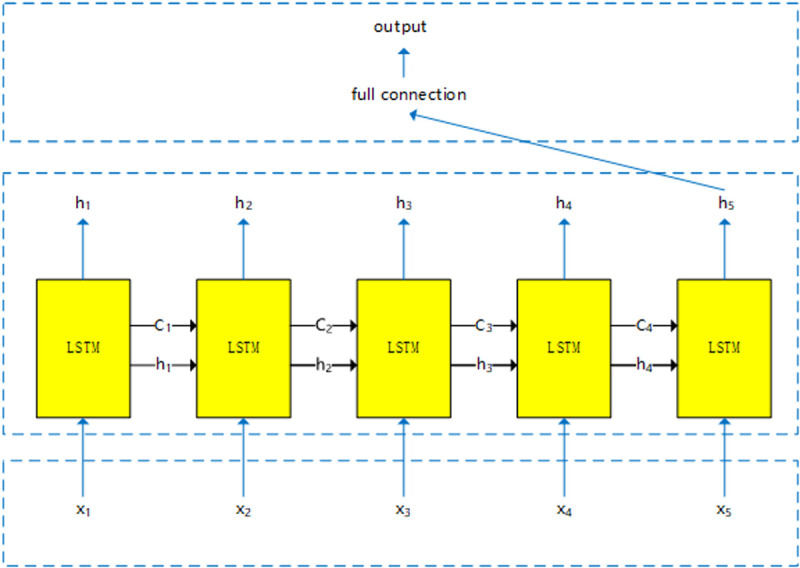
LSTM network overall structure.

Taking an input length of 5 as an example, Fig 4 illustrates the overall architecture of the LSTM network, which comprises five LSTM units structured as depicted in Fig 2. The input data consists of logging curves from five adjacent measurement points. Information from each LSTM unit is sequentially propagated to the next unit, culminating in a fully connected layer at the final stage, where all data is integrated for output. Unlike traditional ANN models, which rely on isolated samples for predictions, the LSTM network facilitates information transfer across sequential samples. This unique capability enhances the network’s ability to capture contextual relationships and improves its overall effectiveness in predicting shear wave velocities across diverse geological formations.

### 2.3 Long Short-term Memory Network Combined with Generative Adversarial Net (GAN-LSTM)

The generative adversarial network (GAN), introduced by Goodfellow in 2014 [2], was initially designed for image generation and enhancement. At its core, the GAN framework operates through a dual-component system: the generator, which guides the creation of data, and the discriminator, which evaluates and corrects discrepancies between the generated data and the target data. This interplay enables the network to produce data that aligns with specific desired characteristics, effectively searching for optimal network parameters under given constraints.

**Fig 5:**
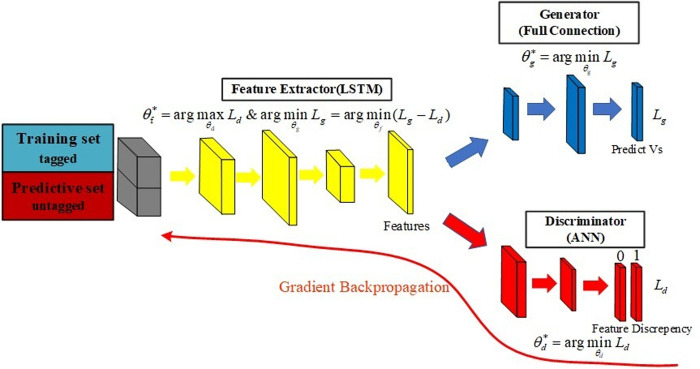
diagram of the GAN-LSTM network. Note: In the Fig 4, $\theta_{f}$, $\theta_{g}$, $\theta_{d}$ are the set of network parameters of feature extractor, the generator and the discriminator respectively; $L_{g}$ and $L_{d}$ are loss functions for the generator and the discriminator.

To address the challenge of accuracy degradation caused by feature distribution mismatches between training and prediction datasets, the GAN-LSTM network is proposed. This hybrid architecture comprises three key components: a feature extractor, a generator, and a discriminator. The feature extractor, implemented as an LSTM network, extracts relevant features from both the training and prediction datasets, which are then fed into the generator and discriminator (Fig 5). The discriminator’s role is to differentiate whether the feature vectors originate from the training or prediction data. When the trained discriminator struggles to distinguish the source of the feature data, it indicates that the LSTM-extracted features are shared across both datasets, thereby mitigating feature bias. Finally, the generator leverages these shared features to predict shear wave velocity, producing results that are better adapted to the prediction dataset.

The mathematical description of the principle of the GAN-LSTM network is given below. Assuming the training set is $D_{s}=\left\{\left(x_{0},y_{0}),...,(x_{i},y_{i}\right)\right\}$, and the prediction set is $D_{t}=\left\{\left(x_{i+1}),...,(x_{i+m}\right)\right\}$. Where, $x\in R^{M*N*O}$ is the independent variables of training data or prediction data, $y\in R^{M*N*O}$ are the corresponding labels. Assuming the training data and the prediction data have the same output space $Y_{s}=Y_{t}$. However, due to the differences between the two data sources, the total feature space or the single feature distribution of the two sets are different ($X_{s}\neq X_{t}$,$P_{s}\left(x_{s}\right)\neq P_{t}\left(x_{t}\right)$), so the conditional probability distribution is also different $Q_{s}\left(y_{s}|x_{s}\right)\neq Q_{t}\left(y_{t}|x_{t}\right)$. The purpose of this study is to use the training data $D_{s}$ along the prediction data $D_{t}$ to learn the migration model *f*, to achieve the target prediction of $y_{t}\in Y_{t}$.

**Fig 6:**
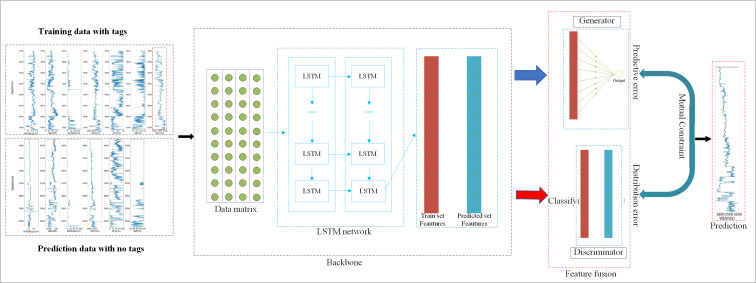
structure of the GAN-LSTM network.

To achieve accurate prediction results, we integrated the LSTM network with a generative adversarial network (GAN-LSTM). The architecture of the GAN-LSTM network is illustrated in Fig 6. The LSTM component serves as the feature extractor, capturing the eigenvectors of the input data. Specifically, the LSTM establishes temporal dependencies and reflections, while the eigenvectors encapsulate the sequence variance and nonlinear relationships derived from the training set. However, these features may not inherently generalize well to the prediction set, making it crucial to minimize the discrepancies between the two datasets.

The discriminator evaluates whether the extracted features originate from the training set or the prediction set. The training set is labeled as 1, and the prediction set as 0, with the discriminator outputting a probability between these two labels. The generator, on the other hand, is guided by the measured shear wave velocity from the training set as its target. The network employs the mean absolute error (MAE) as the generator’s loss function and the binary cross-entropy (BCE) as the discriminator’s loss function.

During training, the generator network aims to minimize the prediction error on the training set, while the discriminator works to reduce the feature distribution differences between the training and prediction sets. This dual optimization ensures that the features used by the generator for shear wave prediction become increasingly similar across both datasets. Therefore, the GAN-LSTM framework achieves a balance between feature alignment and training accuracy, enhancing its generalization capability.

The generator loss function is expressed as:


$$L_{g}=\sum_{i=1}^{n}\left|y_{i}-{\hat{y}}_{i}\right|$$ (7)

The discriminator loss function can be shown as follows:


$$L_{d}=-N/\sum_{i=1}^{N}d_{i}\cdot \log(p(d_{i}))+(1-d_{i})\cdot \log(1-p(d_{i}))$$ (8)

Finally, we use the multi-task neural network optimization strategy [35] to integrate the above loss functions:


$$L=\frac{1}{2\sigma_{1}^{2}}L_{g}+\frac{1}{2\sigma_{2}^{2}}L_{d}+\log\sigma_{1}\sigma_{2}$$ (9)

Where, n is the number of the training set, N is the number of the total samples, $y_{i}$ is the measured value of shear wave, ${\hat{y}}_{i}$ is the predicted value, $d_{i}$ is the label 0 or 1, $p(d_{i})$ is the correct probability; $\sigma_{1}$, $\sigma_{2}$ respectively represent the standard deviation of $L_{g}$, $L_{d}$.

## 3. Data collection and preparation

### 3.1 Data description and processing

The logging data used in this study are from two different drilled wells, Well A and Well B, in Yinggehai Basin, South China Sea. Data from the two wells included seven standard logs: shear velocity (Vs), P-wave velocity (Vp), gamma ray (GR), volume of clay (V clay), density (RHOB), water saturation (SW), and resistivity (RS). These drilling data (logging curves) are used for geological information detection and oil and gas development. The buried depth of the main gas reservoir of Well A is about 4600m ~ 5000m, mainly low permeability sandstone, and the reservoir type is the delta sandstone. The main gas reservoir of Well B is about 4050m ~ 4200m with an apparent gas structure and the reservoir type is submarine fan sandstone. To verify the effectiveness of the proposed GAN-LSTM network, Well A and Well B are divided into the training well and prediction well, respectively. Data from well A are used for training, and data from well B is used for prediction. The statistical indicators of the logging curves are shown in Table 2. The depth of Well A ranges from 3302m to 5042m, the sampling interval is 0.125m, and a total of 13924 samples were measured. The depth of Well B ranges from 3335m to 4076m, and the total sampling points are 5924.

**Table 2:** Statistical characteristics of variables of two wells.

Well name	Statistical index	Vp(m/s)	GR(API)	RHOB(g/cm^3^)	SW(%)	V clay(%)	RS(Ω·m)	Vs(m/s)
Well A	Mean	4221.25	74.31	2.62	0.91	0.61	11.19	2435.39
	Std	430.95	17.01	0.07	0.15	0.31	14.96	345.11
	Min	3008.24	29.17	0.08	0.30	0.01	0.91	1634.64
	max	5599.93	138.43	2.85	1.00	0.99	25.36	3302.90
Well B	Mean	3882.78	107.26	2.60	0.92	0.45	4.88	2191.43
	Std	450.95	23.05	0.09	0.19	0.29	6.68	344.60
	Min	2766.46	37.85	2.11	0.22	0.01	0.85	1498.71
	max	6221.81	168.63	3.41	1.00	1.00	15.78	3199.33

**Fig 7:**
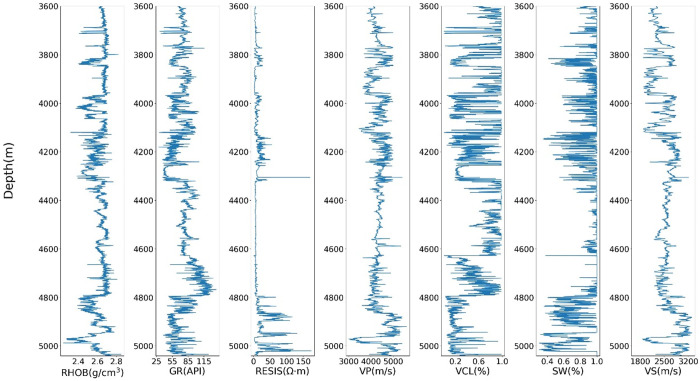
Scatter diagram of logging curves in Well A.

**Fig 8:**
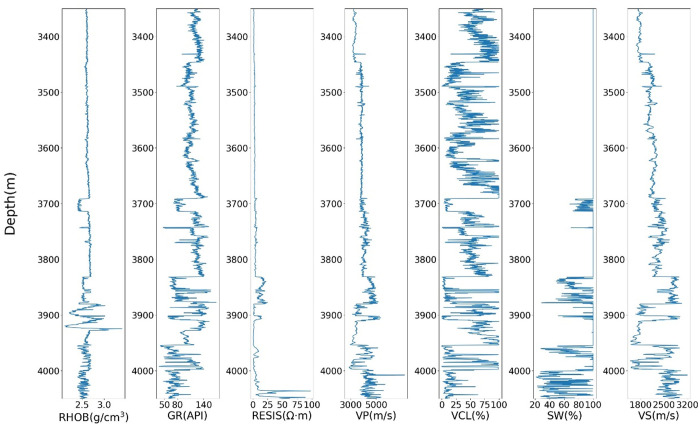
Scatter diagram of logging curves in Well B.

Table 2 is the statistical index description of the conventional logging data of two Wells in the working area. The log curves of two wells are shown in Figs 7 and 8, respectively.

To suit the training of the network, we apply the following reversible transformation to standardize the data:


$$Y=\frac{x-u}{\sigma }$$ (10)

Where, u and σ represent the mean value and standard deviation of the logging curve, respectively. Y is the normalization of x. The mean and standard deviation can be used to restore normalized data to the original data. The original distribution features of the processed data are saved, and the range of the data is narrowed.

**Fig 9:**
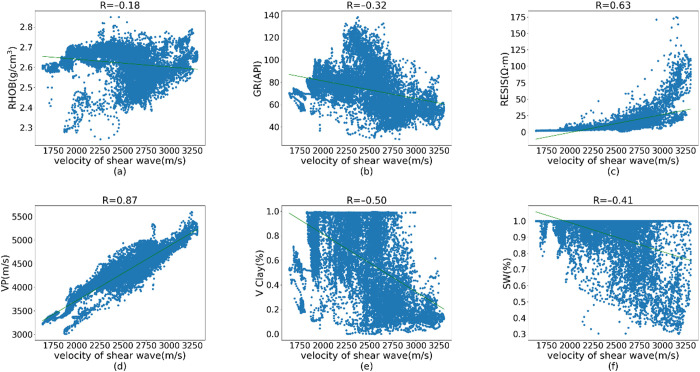
Correlation of logging curves with shear wave velocity.

Various logging attributes exhibit correlations with shear wave velocity. For instance, an increase in water saturation (SW) may result in a decrease in shear wave velocity, while volume of clay (VCL) and gamma ray (GR) can reflect the clay content within the formation. Additionally, the ratio of compressional wave velocity (VP) to shear wave velocity (VS) tends to show a slight increase in shale-rich strata. Furthermore, both compressional and shear wave velocities are commonly influenced by factors such as rock matrix composition, porosity, and the inclusions. Resistivity logs, on the other hand, provide insights into the characteristics of pore fluids within the subsurface. Collectively, these logging curves demonstrate intrinsic relationships not only with each other but also with shear wave velocity. To statistically analyze these relationships, we present cross-plots of each logging curve from Well A against shear wave velocity, as illustrated in Fig 9.

**Fig 10:**
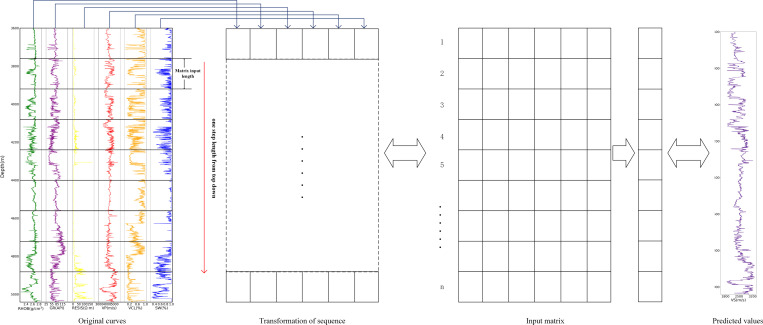
Logging sequence conversion.

Fig 10 illustrates the workflow for transforming logging curves into input matrices. Six logging curves are sampled using a fixed window length to capture actual data points. These data points are then sequentially organized into sample matrices, moving from top to bottom with a step size of 1. Each sample matrix is paired with the corresponding shear wave velocity value at the final time point within the matrix, serving as the label for training. Once the model is trained completely, it is applied to the prediction set to generate the complete shear wave velocity prediction curve.

### 3.2 Parameter settings and algorithm flowchart

The network structure parameters, including the number of hidden layers and hidden units, are determined through an iterative trial-and-error process. Initially, the number of hidden units is fixed, and the number of hidden layers is incrementally increased until a structure is identified where further layers do not significantly reduce the loss value. Subsequently, the number of hidden neurons is adjusted within the determined number of layers to achieve a stable loss value. The specific parameters of the proposed GAN-LSTM network are detailed in Table 3.

**Table 3:** The GAN-LSTM network parameters.

Name of structure	Numbers of input	layers	hidden units	Activate function	Numbers of output	optimizer	LR
LSTM network	6 × 7	2	10	Tanh、Sigmoid	10	Adam	0.001
Generator (Full connection)	10	1	12	Sigmoid	1		
Discriminator(ANN)	10	4	8	Relu	2		

In this architecture, the LSTM network serves as the feature extractor and comprises a 2-layer LSTM network with 10 hidden units. It takes as input six different logging attributes from seven adjacent samples and outputs 10 neurons representing distinct features. The generator consists of a single layer with 12 hidden units. Its role is to transform the feature vectors extracted by the LSTM into predicted values. Parallel to the generator, the discriminator is structured as a 4-layer artificial neural network (ANN) with 8 hidden units. It evaluates the discrepancy between the feature vectors extracted from the training set and the prediction set. As the discriminator’s loss decreases, the feature variables extracted by the LSTM network increasingly capture the shared characteristics of both datasets. This ensures that the generator’s predictions are better generalized to the prediction set, ultimately enhancing the model’s performance.

**Fig 11:**
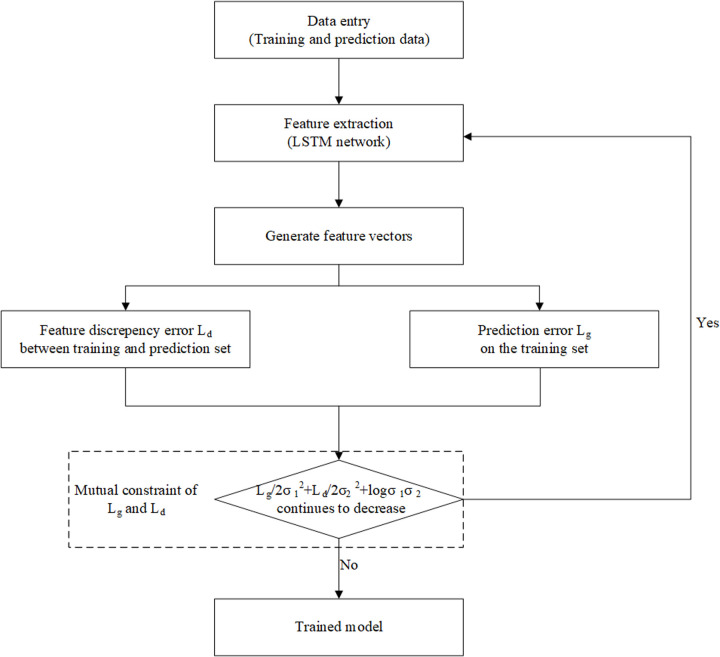
Flowchart of the algorithm.

After configuring the network parameters, the workflow of the GAN-LSTM network (Fig 11) is designed to estimate shear wave velocity. The process begins with the LSTM network extracting and generating critical feature vectors for shear wave prediction. These feature vectors are then utilized to simultaneously minimize prediction errors on the training set and reduce feature discrepancies between the training and prediction sets. The detailed procedure is as follows: First, the LSTM network extracts features from the input data. These features are subsequently fed into both the discriminator and the generator, where the respective loss functions, $L_{d}$ and $L_{g}$, are calculated, respectively. $L_{d}$ aims to minimize the differences between the feature distributions of the two datasets, while $L_{g}$ ensures that the predicted values closely approximate the actual values on the training set. The overall loss L balances these two objectives, enabling the network to predict shear wave velocity effectively while maintaining feature consistency across datasets.

### 3.3 Evaluation metrics

R^2^ and MAE are selected as evaluation metrics to measure the quality of the method involved in this paper. R^2^ indicates the correlation between the predicted value and the measured value and judges whether there is an excessive deviation between them. MAE is the mean absolute error between the predicted value and the measured actual value, which can evaluate how close the predicted value is to the measured value. The formula for R^2^ and MAE can be expressed as follows:


$$MAE=\frac{1}{n}\sum_{i=1}^{n}\left|m_{i}-{\hat{m}}_{i}\right|$$ (11)

$$R^{2}=1-\frac{\sum_{i=1}^{n}{\left({\hat{m}}_{i}-m_{i}\right)}^{2}}{\sum_{i=1}^{n}{\left(m_{i}-\bar{m}\right)}^{2}}$$ (12)

Where, ${\hat{m}}_{i}$ is the estimated value by the prediction method, $m_{i}$ is the measured shear wave velocity, $\bar{m}$ is the mean of measured values, and *n* is the number of predicted samples.

## 4. Results and discussion

### 4.1 Experiment 1

To find out the difference between the LSTM network and the GAN-LSTM network on the prediction set, we selected two point with the same measured value in two wells as a comparison reference for the applied method. The first point is located at the 2092nd sample point of Well A with a depth of 3527m, and the value is 2092.36m/s. The second one is the 1315th sample point of Well B with a depth of 3500m, and the value is 2092.58m/s. While the LSTM network and GAN-LSTM network are used to predict these two points, five feature vectors generated for prediction are shown in Table 4. The Euclidean squared distance of sample point 1 and sample point 2 are calculated. The results generated by the two methods are 0.52 and 0.04 respectively. That shows the GAN-LSTM network can effectively reduce the distance between the eigenvectors of the training set and the prediction set.

**Table 4:** Feature vectors generated by LSTM and GAN-LSTM.

Method	Sampling point	F1	F2	F3	F4	F5
LSTM	point 1	−0.16	−0.26	0.44	−0.18	−0.49
	point 2	0.04	−0.03	0.09	−0.46	−0.01
GAN-LSTM	point 1	−0.61	0.12	−0.29	0.63	−0.63
	point 2	−0.67	0.152	−0.38	0.61	−0.46

The experimental results demonstrate that the proposed GAN-LSTM network can generate highly similar feature vectors between the prediction set and training set while maintaining training accuracy, thereby significantly enhancing prediction generalization capability. To comprehensively evaluate the performance of our proposed network, we conducted comparative studies using both ANN and LSTM networks as benchmarks. The ANN architecture consists of four layers with ten hidden units per layer, while the LSTM network employs a two-layer structure with twelve neurons in each layer and an input sample length of seven. The detailed parameter configuration of the GAN-LSTM network is presented in Table 2. All networks were optimized using the Adam optimizer for parameter fine-tuning.

**Fig 12:**
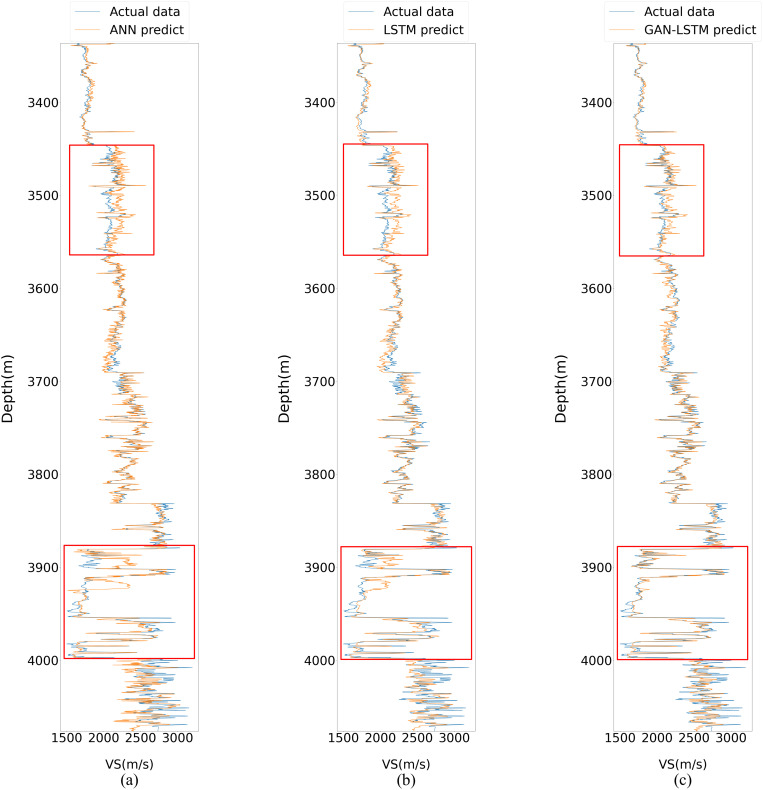
Predictive results of three neural networks in well B.

Fig 12 illustrates the prediction performance of the three networks on the prediction set after being trained with Well A data. While all three methods demonstrate reasonable capability in capturing the general trend of the actual values, the GAN-LSTM network exhibits superior performance with prediction results that are substantially closer to the measured curve, particularly in the depth intervals near 3500m and 3900m, where its advantages are most pronounced.

The training specifications for both LSTM and GAN-LSTM networks are detailed in Table 5. It should be noted that the testing time, which represents the application of trained model parameters to generate predictions from input variables, is relatively brief and thus negligible. The GAN-LSTM architecture incorporates an additional discriminative network component based on ANN structure, resulting in a greater number of trainable parameters compared to the standard LSTM network, consequently requiring longer training times. All training procedures are conducted using PyTorch 1.10.2, with hardware configuration comprising an NVIDIA GeForce RTX 3050 Ti GPU and an Intel Core i5 CPU.

**Table 5:** Network deployment environment and training time.

Equipments	Name	Structure	Optimizer	Parameters	Time
pytorch 1.10.2 RTX 3050 Ti Intel Core i5	LSTM	LSTM cells	Adam	1754	5h37′40″
		Generator(full-connection)			
	GAN-LSTM	LSTM cells	Adam	1968	6h23′56″
			Generator(full-collection)		
			Discriminator(ANN)		

(Mark the upper and lower parts of the curve in red for comparison)

As illustrated in the red frame of Fig 12, the prediction results demonstrate a progressive improvement in consistency with the actual curve from sub Fig (a) to (c). Notably, sub Fig (c) exhibits superior performance in handling areas with significant fluctuations compared to sub Figs (a) and (b). Furthermore, at the 3700m mark, both sub Figs (a) and (b) show slightly inferior performance relative to sub Fig (c), potentially indicating a substantial divergence in characteristics between the training and prediction datasets. Overall, the predictive performance of sub Fig (c) is markedly superior to that of the preceding two subFigs.

**Fig 13:**
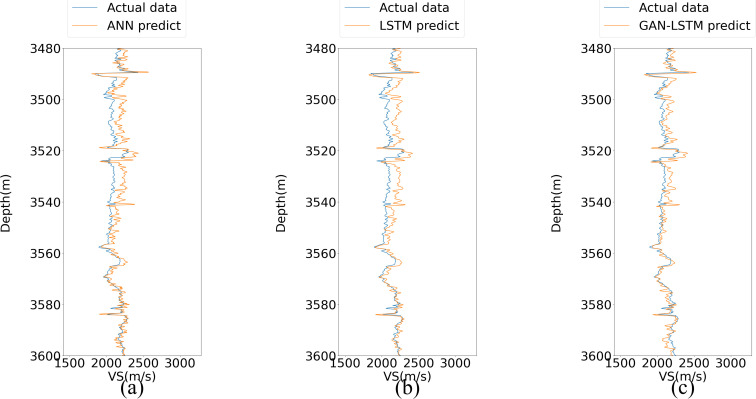
Local amplification comparison in Well B (the upper part marked in Fig 12).

**Fig 14:**
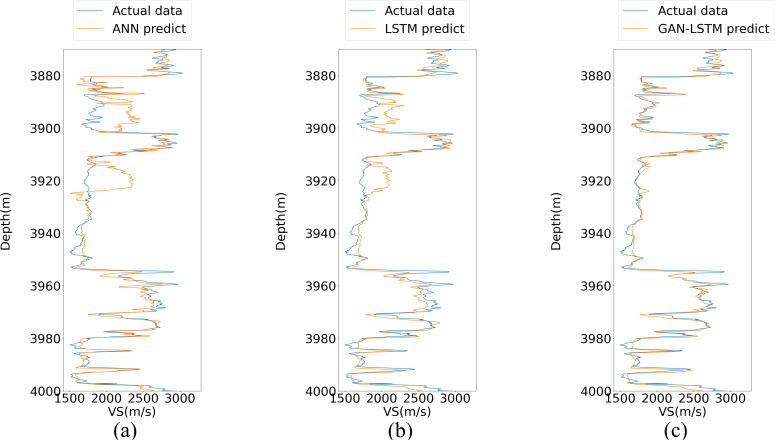
Local amplification comparison in Well B (the lower part marked in Fig 12).

Neural networks have demonstrated their effectiveness in predicting shear wave velocity with minimal deviation. To further illustrate this, two specific intervals are highlighted in red for detailed comparison, as shown in Figs 13 and 14. The results clearly indicate that the predictions generated by the GAN-LSTM model align more closely with the true values compared to those from ANN or LSTM. This improvement is attributed to the GAN-LSTM’s ability to reduce the disparity between prediction and training feature vectors, thereby significantly enhancing prediction accuracy. Moreover, Fig 14 reveals that the sequence predicted by the proposed GAN-LSTM model follows the actual data more closely, even in regions where the curve exhibits significant variations. This underscores the importance of extracting common essential features in addressing the accuracy decline caused by feature discrepancies. By incorporating the discriminator component, the GAN-LSTM network effectively mitigates the influence of non-generalizable features on the prediction set, achieving remarkable predictive performance.

A comparative analysis of the prediction results from (a) to (c) reveals a progressive improvement in the fitting accuracy to the actual curve. Specifically, the prediction curve in (b) demonstrates superior smoothness and adaptability compared to (a), which can be attributed to the LSTM network’s capability to capture contextual information within the sample length. More significantly, the GAN-LSTM network in (c) effectively mitigates the bottleneck effect caused by the characteristic discrepancy between training and prediction datasets during model application. This enhanced performance stems from the network’s ability to simultaneously account for contextual relationships and address uneven feature distribution, making it particularly suitable for practical forecasting scenarios.

**Fig 15:**
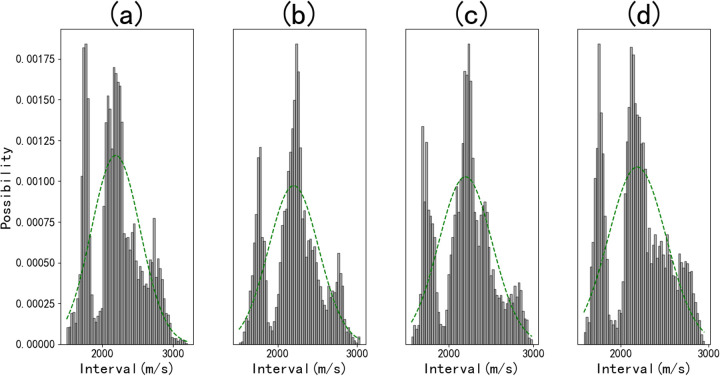
Comparison of the probability distribution of the three prediction methods.

Fig 15(a) presents the frequency histogram of the actual VS curve, while Figs 15(b)–15(d) illustrate the frequency distributions of the prediction results obtained from ANN, LSTM, and GAN-LSTM, respectively. A comparative analysis reveals that the GAN-LSTM prediction results demonstrate remarkable similarity to the actual distribution curve in Fig 15(a), particularly in the velocity ranges around 1800 and 2500. This observation provides substantial evidence that GAN-LSTM effectively adapts to the characteristic distribution of the actual data, outperforming the other two methods in achieving a more accurate alignment between predicted and actual distributions.

**Fig 16:**
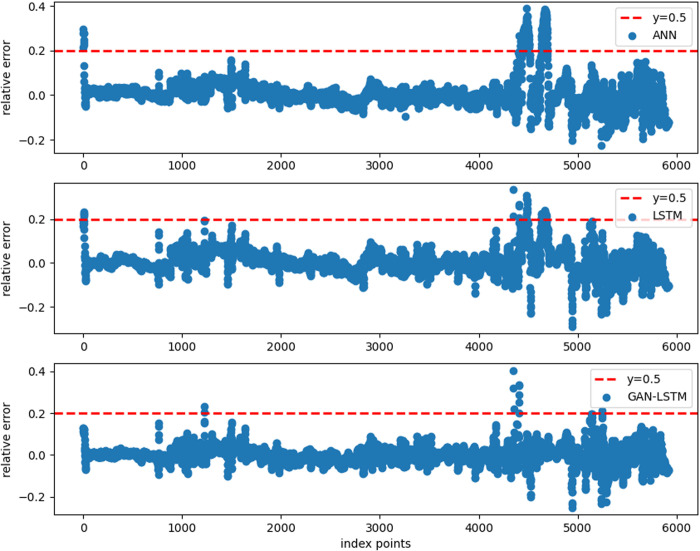
The relative error of the three forecasting methods.

Fig 16 presents a comparative analysis of relative errors generated by the three methods, with the scatter plots (from top to bottom) representing the ratio of predicted deviations to actual values. The ANN results in sub Fig (a) exhibit a substantial number of data points with relative errors exceeding 0.2, which can be attributed to its inability to capture contextual relationships among multiple sample points. In contrast, sub Figs (b) and (c) demonstrate that GAN-LSTM produces significantly less fluctuation, thereby enhancing its practical application value.

**Fig 17:**
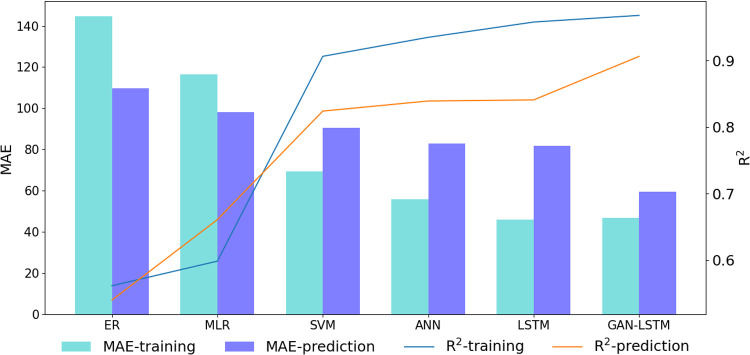
MAE different methods in well B.

For quantitative performance evaluation, we employed MAE (Mean Absolute Error) and R² (Coefficient of Determination) metrics to assess the accuracy of the methods discussed in the literature review. Table 6 provides a comprehensive quantitative comparison of prediction results for both the training well (Well A) and prediction well (Well B) across different methods. Each method was trained on Well A and subsequently applied to Well B for prediction. The analysis reveals that while the LSTM network achieves the lowest MAE (45.87) in the training well, marginally better than GAN-LSTM’s 46.84, the GAN-LSTM network attains the highest R² value of 0.968. More importantly, in the prediction well, GAN-LSTM demonstrates superior performance with MAE and R² values of 59.41 and 0.906, respectively. These results collectively indicate that the proposed network in this study significantly outperforms other methods in both prediction precision and generalization capability. The comparative performance metrics of various methods in the prediction well are visually presented in Fig 17.

**Table 6:** Comparison of accuracy under different methods in two wells.

Well name	Evaluation index	Empirical formula	Multiple regression	SVM	ANN	LSTM	GAN-LSTM
Well A	MAE	144.7485	116.5853	69.4965	55.69	45.87	46.8421
	R^2^	0.5613	0.5983	0.9065	0.935	0.958	0.9681
Well B	MAE	109.6852	98.0144	90.4769	82.79	81.88	59.4101
	R^2^	0.5397	0.6608	0.8241	0.839	0.840	0.9064

As evidenced by Fig 17, the GAN-LSTM model demonstrates superior performance in Well B, exhibiting significantly lower MAE and higher R² values compared to other methods. While the performance difference between LSTM and GAN-LSTM in Well A appears marginal, the GAN-LSTM network achieves substantially higher prediction accuracy in the prediction well. These results conclusively demonstrate that the GAN-LSTM architecture significantly enhances the prediction accuracy of actual shear wave velocity measurements. This improvement can be attributed to the network’s unique capability to utilize the prediction set’s independent variables to minimize the feature distance from the training set, thereby effectively reducing prediction inaccuracies caused by feature distribution dissimilarities.

The quantitative analysis in Fig 17 reveals that GAN-LSTM achieves optimal performance metrics, with the highest R² and lowest MAE values among all methods. Notably, the prediction results of GAN-LSTM show the closest alignment with actual values, as evidenced by the minimal discrepancy between the R² and MAE values of the prediction set and those of the training set. This close correspondence between prediction and training set metrics further confirms the superior generalization capability of the GAN-LSTM approach compared to conventional methods.

**Fig 18:**
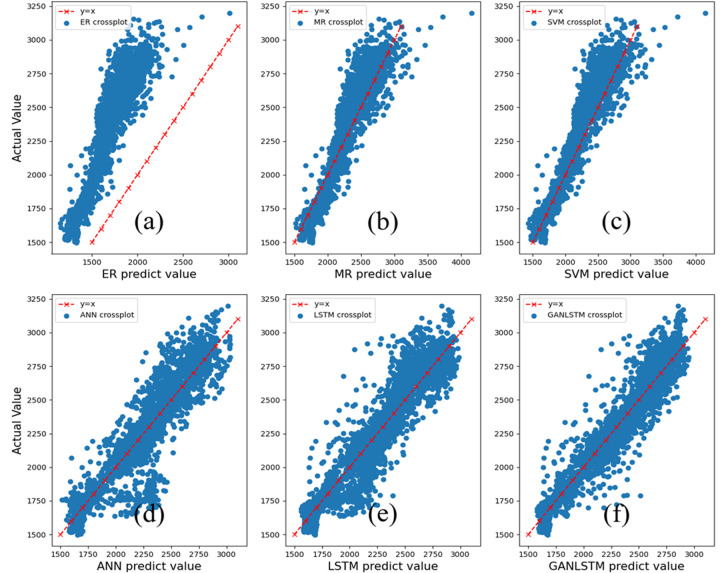
Cross plots of different methods.

Fig 18 demonstrates that the prediction results of the GAN-LSTM method exhibit a significantly tighter clustering around the y = x line, corresponding to its optimal performance metrics of the lowest MAE and highest R² values (as presented in Table 5). This distribution pattern strongly indicates an exceptional consistency between the network’s predicted values and the actual measurements. The GAN-LSTM predictions maintain remarkable agreement with actual values across all depth intervals, with no observable dispersion or disorder in the predicted data points. In contrast, other methods display more scattered data points, suggesting potential prediction biases at various depth levels. This dispersion pattern may reflect underlying characteristic discrepancies between the prediction data and training data, which the GAN-LSTM network appears to effectively mitigate through its advanced architecture.

**Fig 19:**
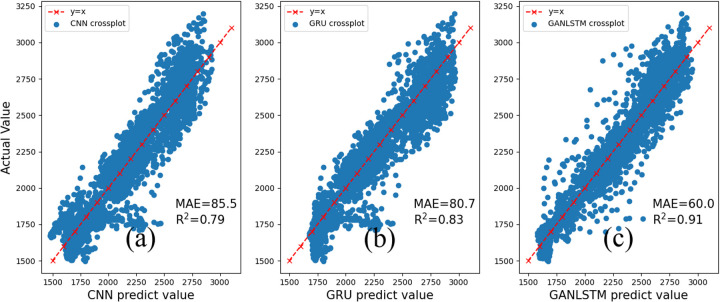
Comparison of CNN [ 35], GRU [36] and GANLSTM.

Fig 19 presents a comprehensive comparison of the proposed GAN-LSTM model with 1DCNN and GRU neural network architectures. The 1DCNN architecture employs a multi-stage feature extraction process, initially projecting the six logging attributes into a 135-dimensional space, followed by the formation of five feature vectors through three one-dimensional convolutional layers (with a kernel length of 3 and stride of 3) for final shear wave velocity determination. The GRU model, while maintaining identical structural parameters to GAN-LSTM (comprising two layers with 10 hidden units each, an input size of 6, and an input length of 7), simplifies the gating mechanism by combining the input and forget gates.

The experimental results reveal distinct performance characteristics among the models. As shown in Fig 19(a), the 1DCNN demonstrates limited capability in extracting contextual information from adjacent samples, resulting in the poorest shear wave velocity prediction performance. Fig 19(b) illustrates that while the GRU model achieves reduced training time through parameter optimization, this comes at the cost of slightly diminished prediction accuracy due to its simplified gating architecture. In contrast, Fig 19(c) demonstrates the superior prediction performance of the proposed GAN-LSTM model on Well B, which achieves the highest prediction accuracy among the three models. This performance advantage underscores GAN-LSTM’s enhanced capabilities in context extraction and feature difference compensation compared to both CNN and GRU architectures.

### 4.2 Experiment 2

**Fig 20:**
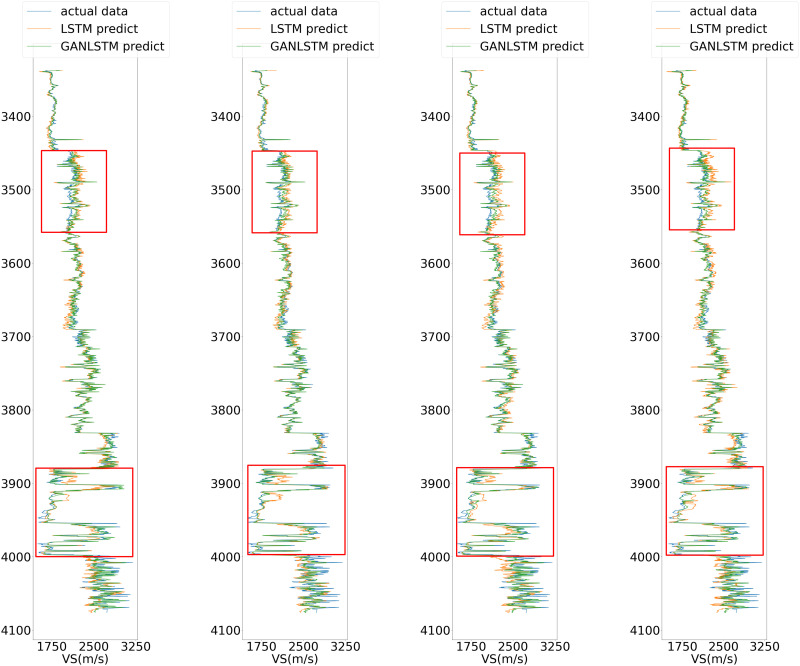
Prediction of LSTM and proposed GAN-LSTM network under different input in Well B. (Input length from left to right is set as 5, 7, 9, 11). (Mark the upper and lower parts of the curve in red for comparison).

To conduct an in-depth analysis of the GAN-LSTM network’s performance, we systematically compared its prediction capabilities with the LSTM network across varying input lengths. This comparative study, when integrated with the findings from Experiment 1, offers a more comprehensive evaluation framework. We examined four distinct input lengths (5, 7, 9, and 11) while maintaining identical network parameters as those used in Experiment 1 for both architectures. The comparative prediction results, illustrated in Fig 20, demonstrate that the GAN-LSTM network achieves significantly enhanced prediction accuracy in Well B across all tested input lengths compared to its LSTM counterpart. This consistent performance improvement not only validates the network’s effectiveness but also highlights its robustness to variations in input sequence length.

**Fig 21:**
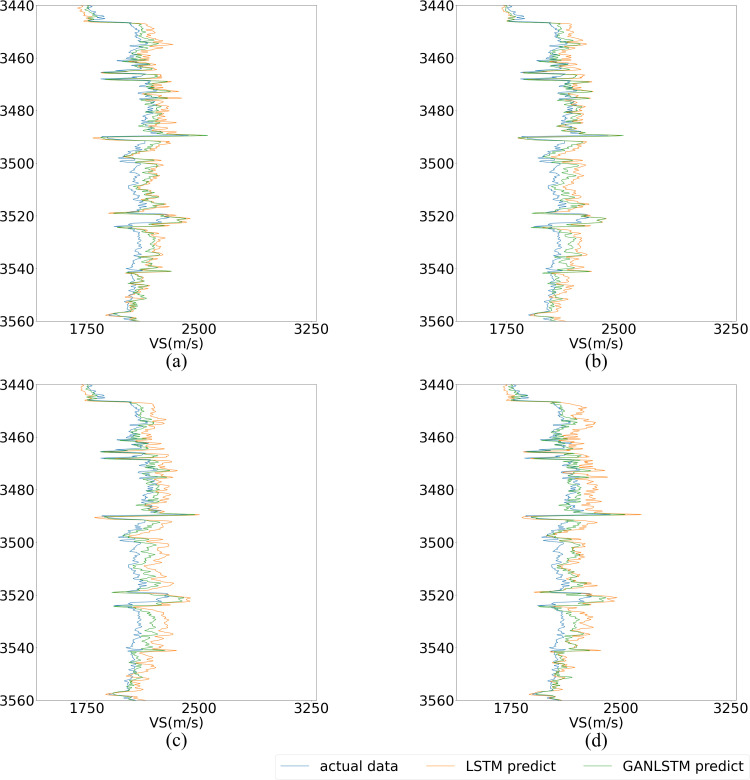
Local magnification of LSTM and GANLSTM’s prediction under different input length. (upper part in Fig 20). (The input length of (a), (b), (c), (d) is set as 5,7,9,11, respectively).

**Fig 22:**
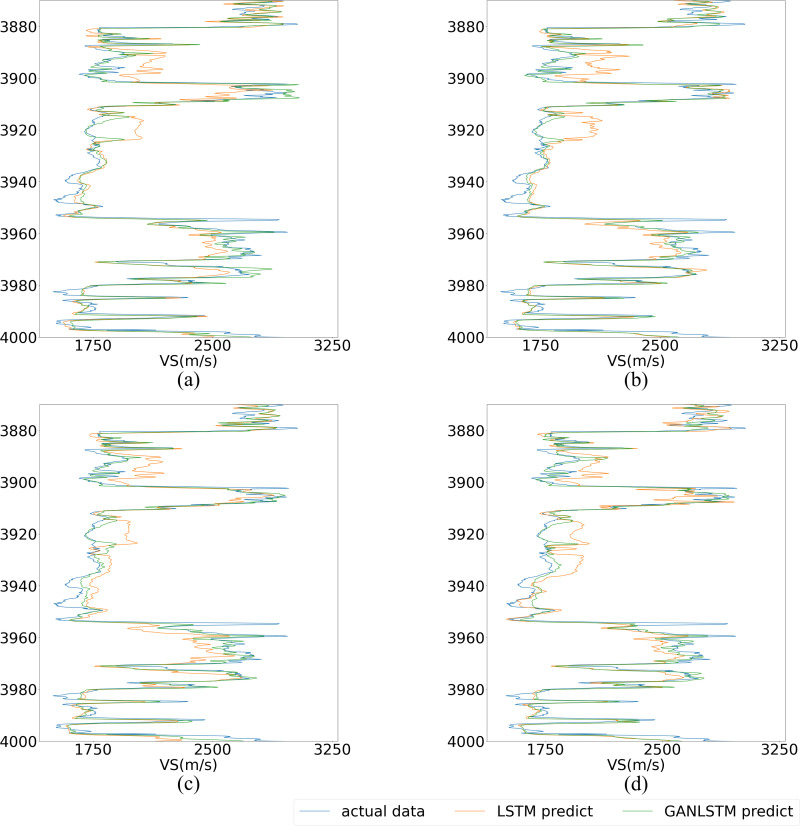
Local magnification of LSTM and GANLSTM’s prediction under different input length. (lower part in Fig 20). (The input length of (a), (b), (c), (d) is set as 5,7,9,11, respectively).

For detailed performance analysis, Figs 21 and 22 present localized comparisons of the marked regions (highlighted in red) from Fig 20. Visual inspection clearly reveals that the GAN-LSTM predictions maintain closer alignment with the true value curve than those of the LSTM network. Notably, as shown in Fig 22, the GAN-LSTM network exhibits superior stability in regions with substantial variations, where the LSTM network’s performance tends to degrade. This comparative analysis substantiates that the proposed method offers both enhanced prediction accuracy and greater stability across diverse geological conditions.

Fig 20 presents a comparative analysis of prediction results from LSTM and GAN-LSTM networks across varying input parameters (with input lengths of 5, 7, 9, and 11 from left to right, respectively). The results within the red boxes (subFigs a-d) reveal that the LSTM predictions (represented by orange curves) exhibit significantly greater deviation from the actual measurements (green curves) compared to other results. Notably, the magnitude of deviation appears positively correlated with the fluctuation intensity of the curves. This phenomenon suggests the existence of feature discrepancies between the training and prediction datasets at certain intervals. The GAN-LSTM network, through its domain transformation mechanism, demonstrates superior capability in addressing these feature inconsistencies, thereby substantially improving prediction accuracy under such challenging conditions.

Figs 21–22 demonstrate that the GAN-LSTM network’s prediction results exhibit superior alignment with the actual curve across various input parameters compared to the LSTM network. While both architectures incorporate contextual information within the sample length, resulting in smooth-adjusted and trend-adapted curves, the GAN-LSTM network significantly outperforms its counterpart by effectively mitigating prediction inaccuracies caused by uneven feature distribution. This enhanced capability to adapt to practical conditions makes GAN-LSTM particularly effective in regions with heterogeneous features.

As illustrated in Fig 20, the comparative analysis of MAE and R² metrics between the two networks under different parameters in the prediction well reveals distinct performance characteristics. Both LSTM and GAN-LSTM demonstrate the fundamental capability to capture the general trend of the actual curve, validating their applicability for shear wave velocity prediction. However, the GAN-LSTM predictions (represented by the orange curve) consistently show closer alignment with actual measurements, particularly in regions with significant variations (Fig 21). This superior performance stems from GAN-LSTM’s ability to reduce the feature discrepancy between prediction and training datasets, resulting in enhanced accuracy and robustness.

The quantitative analysis presented in Table 7 provides further evidence of GAN-LSTM’s advantages. While both methods show comparable accuracy in Well A (training well), GAN-LSTM achieves significantly better R² and MAE metrics in Well B (prediction well) across all tested input lengths. These results collectively demonstrate that GAN-LSTM maintains the prediction accuracy of the training set while substantially improving the performance on the prediction set. The network’s consistent superiority under varying input lengths confirms its enhanced generalization capability and practical applicability in shear wave velocity prediction tasks.

**Table 7:** Evaluation of prediction in Well B under different input window length.

Method	Evaluation index	Length 5	Length 7	Length 9	Length 11
LSTM	MAE	84.8534	81.883	95.8662	89.4747
	R^2^	0.7994	0.8409	0.8174	0.7891
GAN-LSTM	MAE	56.4384	59.4101	54.9613	56.2564
	R^2^	0.9376	0.9064	0.9096	0.9125

**Fig 23:**
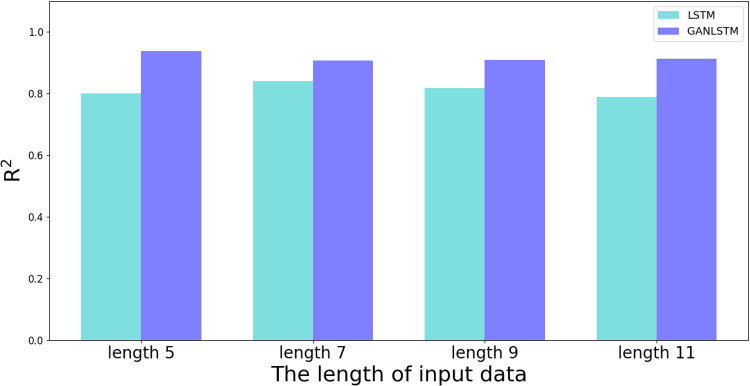
Comparison of R^2^ of results between LSTM and GANLSTM.

**Fig 24:**
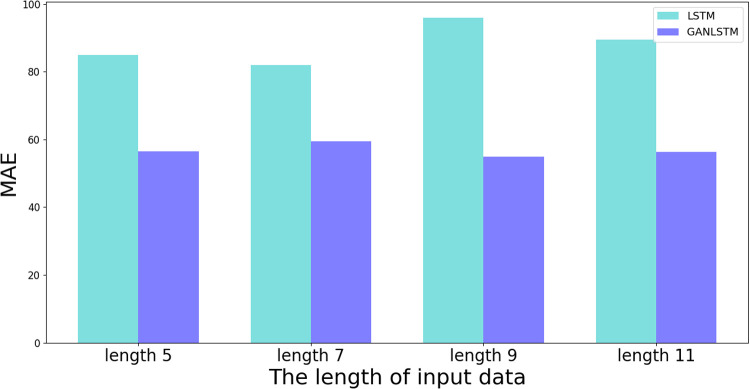
Comparison of MAE of results between LSTM and GANLSTM.

Figs 23 and 24 present column graph visualizations derived from Table 6, facilitating a comprehensive comparison of LSTM and GAN-LSTM performance across different input parameters during prediction. Fig 23 reveals that the proposed GAN-LSTM method consistently achieves higher R² values than LSTM across all tested input lengths, indicating superior prediction consistency. Complementary to this finding, Fig 24 demonstrates that GAN-LSTM maintains lower MAE values compared to LSTM for various input configurations. These quantitative comparisons lead to two significant conclusions: first, the proposed network achieves substantially reduced MAE in prediction; second, it demonstrates markedly higher R² values than the conventional LSTM network.

The experimental results collectively indicate that the GAN-LSTM architecture significantly enhances prediction accuracy across diverse input lengths, thereby ensuring both effectiveness and stability in practical applications. The graphical comparisons clearly demonstrate that regardless of sample input variations, the proposed network maintains superior performance metrics, with consistently lower MAE errors and higher R² values than its LSTM counterpart. This performance advantage stems from the network’s dual capability: not only does it effectively capture contextual information within the curves, but it also addresses the critical issue of feature distribution discrepancies between training and prediction sets. This comprehensive approach significantly enhances the model’s generalization capability, making it particularly suitable for practical situations.

While this study has made significant progress in addressing the feature discrepancy between training and prediction sets, several limitations and areas for future improvement warrant attention:

First, the parameters within the deep learning network for shear wave velocity prediction lack explicit physical interpretation, and their actual significance in the final model remains unclear. This interpretability gap represents a fundamental challenge in applying deep learning to geophysical problems. Second, the integration of theoretical mechanisms into the shear wave velocity prediction framework merits further investigation, as such incorporation could potentially enhance both model performance and physical plausibility. Third, emerging deep learning paradigms, such as instance learning [37,38] and other advanced architectures, may offer novel approaches to address current limitations.

Although this research represents an advancement over traditional LSTM through substantial modifications, the core architecture remains fundamentally similar. To better meet the demands of production practice, future work should focus on developing innovative shear wave prediction models that not only improve accuracy but also enhance interpretability and physical consistency. These advancements could potentially lead to more reliable and practical solutions for shear wave velocity prediction in various geological contexts.

## 5. Conclusion

Shear wave velocity logging holds significant position in petroleum exploration. To address the scarcity of actual VS curves, we have developed a neural network-based method for high-precision prediction of shear wave curves. Through comprehensive literature review and experimental analysis, we have identified that LSTM networks possess superior capability in extracting environmental context information, making them more suitable for time-varying sequences compared to ANN, thereby achieving higher prediction accuracy. However, logging data characteristics exhibit inherent variability across both temporal (depth) and spatial (well location) dimensions. To address the feature discrepancy between training and prediction sets, we propose a novel architecture combining long short-term memory networks with generative adversarial networks (GAN-LSTM). This approach demonstrates superior performance, with similar frequency distributions to actual curves and exhibiting lower relative errors and higher consistency compared to other neural network methods. Experimental results substantiate these advantages: in Experiment 1, GAN-LSTM achieves optimal metrics in the prediction well (MAE = 59.4101, R² = 0.9064), surpassing all comparative methods. Experiment 2 further demonstrates the network’s robustness, showing significantly better prediction results across various input lengths compared to conventional LSTM networks. Notably, GAN-LSTM exhibits exceptional performance in regions with substantial geological variations. These findings collectively demonstrate that the proposed network architecture successfully extracts essential features and significantly enhances practical application accuracy in shear wave velocity prediction.

## Supporting information

S1 Data_wellA&wellB.xlsx. The part of Information Data. (XLSX)

## Acronym:

LSTM: Long Short-Term Memory Network
ANN: Artificial Neural Network
SVM: Support Vector Machine
GAN-LSTM: Long Short-Term Memory Network with Generative Adversarial Mechanism
RMSE: Root Mean Square Error
MAE: Mean Absolute Error
R^2^: Coefficient of Determination
ER: Empiral Relation
